# Effectiveness of remote monitoring in improving CPAP compliance and the impact of preexisting organisation of standard care: a randomised controlled trial

**DOI:** 10.1007/s11325-024-03042-z

**Published:** 2024-05-16

**Authors:** Stephan van der Kleij, Ingrid de Backer, Barbara Hanraets, Johan Verbraecken, Jerryll Asin

**Affiliations:** 1 Department of Pulmonary Medicine, Centre for Sleep Medicine, Amphia Hospital, Postbus 90158, 4800 RK Breda, The Netherlands; 2 Centre for Home Ventilation, Erasmus MC, Postbus 2040, 3000 CA Rotterdam, The Netherlands; 3 Department of Pulmonary Medicine, Centre for Home Ventilation, UMC Utrecht, Utrecht, The Netherlands; 4 Multidisciplinary Sleep Disorders Centre, Antwerp University Hospital and University of Antwerp, Edegem, Belgium

**Keywords:** Obstructive sleep apnea, Telemedicine, Telemonitoring/remote monitoring, Compliance, Positive airway pressure

## Abstract

**Purpose:**

Continuous positive airway pressure (CPAP) is often the treatment of choice for obstructive sleep apnea (OSA). Short-term adherence and early perceived benefits are the best predictors of long-term adherence. The aim of this study was to determine the effect of telemonitoring in the first period of treatment with CPAP (auto-titrating PAP) on compliance and the long-term outcome.

**Methods:**

Patients aged between 18–75 years old with symptomatic severe OSA (apnea–hypopnea index (AHI) ≥ 30) requiring CPAP therapy were included in this single-blind, single-centre, randomised, controlled trial. They were divided into 2 groups (telemonitored standard clinical care versus standard clinical care without telemonitoring).

**Results:**

A total of 230 patients (115 patients/group) were included (mean age 54 ± 16.6 years, BMI 32.6 ± 5.4 kg/m^2^, ESS 13.1 ± 6.2, AHI 47.5 ± 14.8/hr). At week 10 compliance was similar in both groups (telemonitoring vs control 6:27 and 6:35 h, respectively, p = 0.57), as were AHI (2.4; 2.4/hr, p = 0.89) and ESS (5.8; 4.9, p = 0.22). The number of contacts of a patient with a healthcare professional was significantly higher during the follow-up from week 3 until week 10 (0.25; 0.13, p = 0.03). The number of patients who could be evaluated after 1 year was equally distributed in both groups (104; 104, p = 1.00), as were compliance (6:43; 6:49 h, p = 0.59) and residual AHI (1.9; 2.2/hr, p = 0.41).

**Conclusions:**

In patients with severe OSA with standard intensive follow-up during the initial weeks of CPAP therapy and good compliance, telemonitoring did not improve CPAP compliance nor the clinical outcome in the short or long term. The practical consequences can be highly relevant for patients and healthcare professionals.

## Introduction

Obstructive sleep apnea (OSA) is the most common sleep-related breathing disorder. OSA can be characterised by partial or complete inspiratory upper airway collapse during sleep, resulting in periodic oxygen desaturation and disruption of sleep [1, 2]. When these events occur frequently, they can result in daytime symptoms such as excessive sleepiness, impaired cognition and memory, mood alterations, and impediment in daily functioning. Patients with untreated OSA are at increased risk for cardiovascular and metabolic complications [3–8].

Continuous positive airway pressure (CPAP) is the treatment of choice for severe OSA, with adherence of 60-70% [9, 10]. Because short-term adherence and early perceived benefits are the best predictors of long-term adherence, efforts to optimise adherence are best done before or shortly after starting treatment [10–14]. Intensive support has proven before to be beneficial [15–17].

Telemedicine can be of use in multiple ways [18]. Telemonitoring is enabling clinicians to continuously monitor specific parameters of treatment efficacy, thereby permitting prompt and timely adjustments to therapy if indicated. This was originally done by interactive patient-controlled communication by telephone combined with positive re-enforcement and later by remote monitoring [12, 19]. The primary outcome of our study was to investigate the effect of telemonitoring on mean hours/night of CPAP use after 10 weeks of treatment.

## Methods

We performed a single-blind, single-centre, randomised, controlled trial of telemonitoring added to standard care versus standard care alone in patients with severe OSA (AHI ≥30). Patients were randomised to the telemonitoring or standard care group in a 1:1 ratio through blinded allocation using an envelope-procedure. The study was approved by a Research Ethics Board of the Maxima Medisch Centrum (NL 52339.015.15).

### Participants

Participants were recruited among adult patients (18 – 75 years of age) with newly diagnosed symptomatic severe obstructive sleep apnea (AHI ≥ 30 /hr by polysomnography (PSG) according to the AASM) with an indication for CPAP therapy. Written informed consent was mandatory. Exclusion criteria were an anxiety disorder, mental disorder or handicap, a significant central sleep apnea component, and expected other changes in physical wellbeing due to non-related comorbidity and/or expected weight loss or gain such as pregnancy and bariatric surgery.

### Protocol

All patients were included after a standard workup. After obtaining permission by written informed consent, patients were randomised to either auto-titrating CPAP (APAP) with remote monitoring (AirView) or APAP without remote monitoring. Patients of both groups were provided with a Resmed Airsense 9 or 10 APAP with an option for telemonitoring and an (oro)nasal mask. Patients were blinded for the group to which they were allocated until there was an unscheduled contact for an intervention. APAP was started and regular check-ups were planned for both groups according to the standard care in our hospital. These included a phone call on day 2 or 3 after the start of treatment and hospital visits at weeks 3 and 10, and after 6 and 12 months. During the check-up at week 10, an evaluation was done with the patient and the decision was taken whether or not to continue APAP therapy. All patients were instructed to contact the clinic in case of problems. The clinic was available on workdays during office hours. In the intervention group, data from the APAP was assessed daily through remote monitoring during the first 3 weeks, except weekends (hours of usage per night, mask leakage and AHI). From week 4 until week 10, data was assessed on a weekly basis (the last night of that week was measured) unless there was an indication to monitor more frequently.

**Fig. 1:**
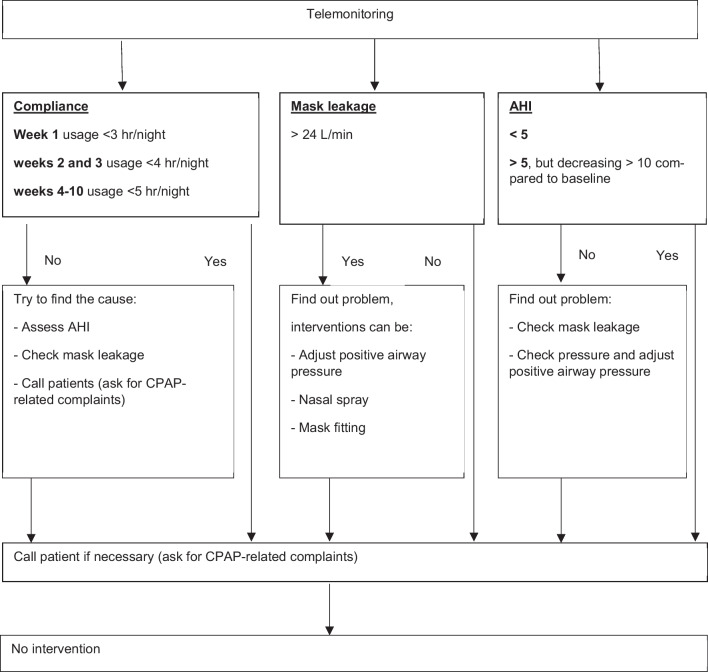
Decision tree for intervention during telemonitoring based on compliance, mask leakage and AHI which could be indicated independent of each other

If CPAP usage was <3 hours per night in week 1, <4 hours per night in weeks 2 and 3, or <5 hours in weeks 4-10, patients were called by the nurse of the outpatient sleep clinic. Assessment included among others mask problems such as airleakage or discomfort, or nasal patency. When necessary, action was taken for the problem such as mask fitting or prescription of nasal spray. An intervention was also performed if the AHI did not decrease to <10/hour, or if a mask leakage of >24L/min was detected (fig. 1).

Once an early intervention was needed, patients knew that they were assigned to the remote monitoring group. From that point on it was no longer a single-blind study. These interventions were documented.

At baseline and after 10 weeks of follow-up, patients filled out the ESS and FSS questionnaires for sleepiness and fatigue, respectively. ESS ≥ 11 and FSS ≥ 36 were considered clinically relevant for sleepiness or fatigue, with higher scores indicating more complaints [20].

We recorded mean hours of CPAP use per night, the number of patients who dropped out, the number of contacts initiated by the patient and interventions initiated by the nurses, for example for CPAP-related complaints (mask leakage) and a persistently high AHI.

### Statistical analysis

The primary outcomes measure was mean hours of CPAP use per night after 10 weeks follow-up. Secondary outcomes were mean hours of CPAP use per night after one year and the number of compliant patients and/or dropouts after 10 weeks and 1 year follow-up. Compliance was defined as a CPAP use > 4 h during at least 5 days a week. We also looked at the efficiency of additional telemonitoring regarding additional check-ups and contacts with the staff related to symptoms or complaints. The analysis was performed by an intention-to-treat protocol. Continuous data were presented as mean ± standard deviation. For the primary outcome (mean hours of CPAP use per night), comparison was made using an unpaired two-sample t-test. The statistical tests were two-sided, and tests with a value of p<0.05 were considered significant.

A variety of secondary outcomes was analysed: the difference in mean hours per night of CPAP use after one year follow-up, and the difference in numbers of patients discontinuing CPAP use and contacts were tested using an unpaired two-sample t-test. A multivariate linear regression analysis was used to assess possible independent predictors of CPAP adherence if applicable.

A pilot study showed a mean compliance of 6:12 hours after 3 months of CPAP-use with a standard deviation of 1:21 hours. Power calculation mandated the inclusion of 230 patients for a difference of 0:30 hours between groups (power 80%, α=0.05, β=0.2) [21].

## Results

**Fig. 2:**
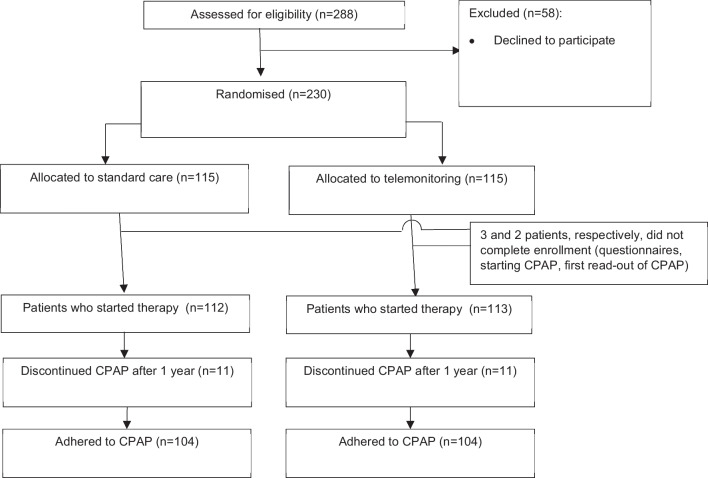
Inclusion flowchart

During the study period, a total of 288 patients was screened for inclusion. A total of 230 patients who were willing and eligible to participate were enrolled in the study with balanced baseline characteristics (fig. 2).

Table 1 shows the patient characteristics. There were no significant differences in age, BMI, AHI, ESS or FSS between the two study groups.

**Table 1:** Baseline characteristics (mean ± SD)

Baseline	Control	Telemonitoring	P
Number of patients at week 3	112	113	0.65
% Male	74	75	0.88
Age [years]	54 (± 17.1)	54 (± 16.1)	0.96
AHI [/hr]	48 (± 14.8)	47 (± 14.7)	0.58
BMI [kg/m^2^]	32.7 (± 5.2)	32.5 (± 5.6)	0.71
ESS	12.3 (± 5.6)	13.8 (± 7.0)	0.09
FSS	37.1 (± 14.1)	37 (± 12.7)	0.95

*Unpaired t-test for continuous variables, Chi-Square test for numbers or proportions*

*AHI: apnea–hypopnea index, BMI: body mass index, ESS: Epworth Sleepiness Scale Score, FSS: Fatigue Severity Scale, SD: standard deviation*

Compliance with CPAP after 10 weeks was similar in both groups. During the last week of this period, patients in the telemonitoring arm used CPAP for a mean of 6:27 hr/day versus 6:35 hr/day in the standard care arm (mean difference = 8 min, p = 0.57) (Table 2). Other outcomes of therapy were comparable as well: AHI (2.4; 2.4/hr, p = 0.89), ESS (5.8; 4.9, p = 0.22), FSS (28.2; 28.7, p = 0.80). The number of contacts of each patient with a healthcare professional was significantly higher during the follow-up from week 3 until week 10 in the telemonitoring group (0.25; 0.13, p = 0.03). The number of patients who could be evaluated after 1 year was equally distributed (104 vs 104, p=1.00), as were compliance in mean hours of CPAP use (6:43; 6:49 hr, p=0.59) and residual AHI (1.9; 2.2/hr, p=0.41).

**Table 2:** Results of telemonitoring and standard care (mean ± SD)

Results		Control	Telemonitoring	P
3 weeks	Compliance [hr] (SD)	6:37 (1:42)	6:18 (1:37)	0.17
	AHI [/hr] (SD)	2.85 (2.62)	3.01 (5.63)	0.79
10 weeks	Compliance [hr] (SD)	6:35 (1:45)	6:27 (1:42)	0.57
	AHI [/hr] (SD)	2.36 (2.43)	2.40 (2,46)	0.89
	ESS (SD)	4.93 (4.48)	5.75 (5.28)	0.22
	FSS (SD)	28.69 (14.90)	28.20 (13.65)	0.80
	Interventions between weeks 3 and 10 (SD)	0.13 (0.33)	0.25 (0.50)	0.03
	Dropouts (%)	6 (5)	6 (5)	1.00
6 months	Compliance [hr] (SD)	6:37 (1:39)	6:33 (1:19)	0.76
	AHI [/hr] (SD)	2.17 (2.62)	2.11 (2.01)	0.83
12 months	Compliance [hr] (SD)	6:49 (1:12)	6:43 (1:20)	0.59
	AHI [/hr] (SD)	2.18 (2.27)	1.93 (2.12)	0.41
	Dropouts (%)	11 (10)	11 (10)	1.00

*Unpaired t-test for continuous variables, Chi-Square test for numbers or proportions*

*AHI: apnea–hypopnea index, ESS: Epworth Sleepiness Scale Score, FSS: Fatigue Severity Scale, SD: standard deviation*

In univariate analyses, there were no significant predictors of CPAP adherence or discontinuation of CPAP (ESS, age).

## Discussion

Remote monitoring of CPAP therapy did not have any additional effect on compliance in our population of CPAP- naïve patients with symptomatic severe OSA in the setting of a patient-counselling system with high adherence to CPAP, which implies a ceiling effect for improving CPAP compliance (90%, 6:46 hours/night after 1 year). This high compliance may be due to a high complaint burden (ESS) and the standard intensive follow-up and support already used in the control group, which also showed good compliance. This is in line with other studies and a recent meta-analysis, which confirmed that telemonitoring was helpful when basal compliance is lower (60-70%) in the control group [18, 22, 23]. Our study is the first one performed in the Netherlands that investigated the effectiveness of remote monitoring in improving CPAP compliance in patients with symptomatic severe OSA. The results from earlier studies in a Canadian and American population cannot be generalised to our Dutch population, especially when a high adherence is expected in the general sleep-apnea population [24, 25].

The interventions in our study were mainly focussed on improving compliance and reducing the side effects of CPAP therapy, using telemonitoring data to guide and adjust the therapy if needed in an early stage. Interventions were predominantly performed during the first 3 weeks. Our study mainly carried out interventions during the first 10 weeks of treatment. Other studies have shown that interventions which were implemented in an early stage had the greatest effect on compliance [26]. It was not clear when interventions are necessary in patients with a high degree of compliance.

Our protocol for interventions used lower thresholds for intervention compared to other studies. Our minimum for compliance was 5 hr/night instead of 3 or 4 hours and mask leakage under 24 l/min instead of 27-50 l/min [18, 27, 28].

Dropouts occurred mainly in the first 3 months of treatment, primarily because of CPAP intolerance, and were equally distributed over both groups. Dropouts after 3 months were mainly due to other reasons, for example resolved OSA (weight loss), OSA-unrelated death (2), metastatic malignancy or change to a mandibular advancement device (MAD).

Turino et al. had similar results to ours using a control reference group with a lower compliance of 4.9 hr/night when compared to our study, but with a lower burden of complaints at baseline [28]. Antallainen et al. reported a comparable adherence level in both groups compared to our study, with a lower level of complaints at baseline but probably a more intense follow-up during the first 4 weeks [AHI 34.6/hr, ESS 8.2]. In their study, adjustments were performed by a nurse by phone or visit to the outpatient ward and not by a web-based approach or by making use of other multimedia, as was done in most other studies. In addition, Antallainen et al. only randomised patients after the titration period, which was done as well by Sparrow et al. [16, 24]. The latter authors suggested that they may have lost patients who had a negative titration experience, or may have had a bias in the enrolment of patients who anticipated they needed help with adherence to CPAP. Our results showed that telemonitoring can be used directly from the initiation of PAP therapy, and dropouts due to intolerance in a highly compliant group could not be influenced by this telemonitoring approach.

Studies with patient groups with low compliance (mean ≤ 3.2 hr/night) such as Sparrow et al. and Fox et al. (self- reported compliance by automated telephone linked motivational enhancement or telemonitoring to interventions by the nurse) showed that an intervention resulted in a clinically and significantly improved CPAP adherence in both their control and telemonitored patient group compared to our study [16, 27]. Criteria for sufficient use are defined as 4 hours of use per night on 70% of days [29]. However, the complaint burden was lower in the study of Fox et al. (ESS before treatment: 9.9 vs 9.7 for remote monitoring vs control) [27].

Bruyneel et al. suggested that guidance by medical staff is more effective in optimising compliance than intervention generated by automated feedback systems for both the telemonitoring and the control group [15]. The limitations in previous studies are the relatively modest number of patients included and the lack of using the same type of CPAP device with only the telemonitoring option switched off in the control group [27]. This implies that the patients’ perception of monitoring might have increased their adherence. They might have felt more responsible and more motivated not to disappoint their healthcare providers. In our study, both groups used the same CPAP device, with the telemonitoring function switched off in the control group.

Our study emphasises the effect of telemonitoring of APAP parameters (such as compliance, air leakage and AHI) on compliance during the first weeks after starting treatment in a Dutch population, as well as the effect of early intervention on continuing the therapy after 10 weeks up to 1 year follow-up. As previously mentioned, the first weeks of treatment are crucial for adherence. Remote monitoring can be helpful during the initial CPAP titration, for example to detect problems earlier, and could reduce the need for outpatient contacts, but there is insufficient evidence that the total number of contacts can be reduced without sacrificing compliance. Remote monitoring may simplify and improve telemedicine, especially for patients who have to travel a long distance to the hospital, and may replace physical appointments or may complement an E-health consultation [24]. Implementation of this type of system could result in substantially improved care in patients with OSA, but this requires the availability of wireless options and internet, depending on the type of remote-monitoring system. Effective therapy with good adherence mostly depends on the patient’s motivation, supplemented with a solid foundation of a well-functioning outpatient clinic providing personal support for the patient with human interaction. Implementation of telemonitoring may also lead to greater patient satisfaction and possibly a reduction in staff time and cost. It is essential to regulate telemonitoring contacts according to a standardised protocol to prevent unnecessary contacts with the patient and temper the workload for the sleep professionals. In our clinic this protocol has been effectively implemented in daily practice according to the conditional recommendation of the American Academy of Sleep Medicine guideline [30].

### Limitations

There are several limitations to our study. Only patients with a high AHI and excessive daytime sleepiness were included. Therefore, the results cannot be extrapolated to patients with less severe OSA. Furthermore, this is a single-centre study, and reproducing our findings in other centres and patient populations would allow generalisability. Our high adherence might be explained by our intensive standard care protocol.

## Conclusions

Remote monitoring of CPAP had no additional effect on compliance in our single-centre population of patients with symptomatic severe OSA. This was probably because of the standard intensive follow-up system and support, with high adherence to CPAP as a result (“ceiling effect”). However, telemonitoring might be of benefit in certain patient groups with complex problems at the start of therapy or observed low adherence. These findings must be verified in other centres. The benefits may extend to patients with less mobility or long traveling distances and can replace outpatient consultations by E-health.

## Data Availability

The datasets generated during and/or analysed during the current study are available from the corresponding author on reasonable request.

## References

[1] Weaver TE, Sawyer AM (2010) Adherence to continuous positive airway pressure treatment for obstructive sleep apnoea: implications for future interventions. Indian J Med Res 131:245–258 20308750 PMC2972705

[2] Kushida A et al (2005) Practice Parameters for the Indications for Polysomnography and Related Procedures: An Update for 2005. Sleep 28(4):299–519 10.1093/sleep/28.4.499 16171294

[3] Kendzerska T et al (2014) Obstructive sleep apnea and incident diabetes. A historical cohort study. Am J Respir Crit Care Med 190(2):218–25 24897551 10.1164/rccm.201312-2209OC

[4] Lacasse Y, Godbout C, Sériès F (2002) Health-related quality of life in obstructive sleep apnoea. Eur Respir J 19(3):499–503 11936529 10.1183/09031936.02.00216902

[5] Weaver T et al (1999) Improvement in affect after 3 mo. CPAP: Multi-center study. Am J Respir Crit Care Med 159(3):A770–A770

[6] Al Lawati NM, Patel SR, Ayas NT (2009) Epidemiology, risk factors, and consequences of obstructive sleep apnea and short sleep duration. Prog Cardiovasc Dis 51(4):285–93 19110130 10.1016/j.pcad.2008.08.001

[7] Marin JM et al (2005) Long-term cardiovascular outcomes in men with obstructive sleep apnoea- hypopnoea with or without treatment with continuous positive airway pressure: an observational study. Lancet 365(9464):1046–1053 15781100 10.1016/S0140-6736(05)71141-7

[8] Weaver TE et al (2007) Relationship between hours of CPAP use and achieving normal levels of sleepiness and daily functioning. Sleep 30(6):711–719 17580592 10.1093/sleep/30.6.711 PMC1978355

[9] Lacassagne L et al (2000) [Results of 248 patients with sleep apnea syndrome treated by continuous positive pressure ventilation between 1990 and 1995. A study of compliance and outcome of the apnea‐hypopnea index] Suivi de 248 patients présentant un syndrome d’apnée du sommeil traités par pression positive continue entre 1990 et 1995. Etude de l’observance et du devenir de l’index d’apnée‐hypopnée. Rev Mal Respir 17(2):467–74 10859765

[10] Reeves-Hoche MK, Meck R, Zwillich CW (1994) Nasal CPAP: an objective evaluation of patient compliance. Am J Respir Crit Care Med 149(1):149–154 8111574 10.1164/ajrccm.149.1.8111574

[11] Aloia MS et al (2007) How early in treatment is PAP adherence established? Revisiting night-to- night variability. Behav Sleep Med 5(3):229–240 17680733 10.1080/15402000701264005

[12] DeMolles DA et al (2004) A pilot trial of a telecommunications system in sleep apnea management. Med Care 42(8):764–769 15258478 10.1097/01.mlr.0000132353.99209.fe

[13] Jordan AS, McSharry DG, Malhotra A (2014) Adult obstructive sleep apnoea. Lancet 383(9918):736–747 23910433 10.1016/S0140-6736(13)60734-5 PMC3909558

[14] Kribbs NB et al (1993) Objective measurement of patterns of nasal CPAP use by patients with obstructive sleep apnea. Am Rev Respir Dis 147(4):887–895 8466125 10.1164/ajrccm/147.4.887

[15] Bruyneel M (2016) Technical Developments and Clinical Use of Telemedicine in Sleep Medicine. J Clin Med 5(12):116 27983582 10.3390/jcm5120116 PMC5184789

[16] Sparrow D et al (2010) A telemedicine intervention to improve adherence to continuous positive airway pressure: a randomised controlled trial. Thorax 65(12):1061–1066 20880872 10.1136/thx.2009.133215

[17] Woehrle H et al (2017) Telemedicine-based proactive patient management during positive airway pressure therapy: Impact on therapy termination rate. Somnologie (Berl) 21(2):121–127 28706464 10.1007/s11818-016-0098-9 PMC5486580

[18] Verbraecken J (2021) Telemedicine in Sleep-Disordered Breathing: Expanding the Horizons. Sleep Med Clin 16(3):417–445 34325821 10.1016/j.jsmc.2021.05.009

[19] Coma-Del-Corral MJ et al (2013) Reliability of telemedicine in the diagnosis and treatment of sleep apnea syndrome. Telemed J E Health 19(1):7–12 23186084 10.1089/tmj.2012.0007 PMC3546414

[20] Johns MW (1993) Daytime sleepiness, snoring, and obstructive sleep apnea. Epworth Sleepiness Scale Chest 103(1):30–36 8417909 10.1378/chest.103.1.30

[21] Wittes J (2002) Sample size calculations for randomized controlled trials. Epidemiol Rev 24(1):39–53 12119854 10.1093/epirev/24.1.39

[22] Aardoom JJ et al (2020) Effectiveness of eHealth Interventions in Improving Treatment Adherence for Adults With Obstructive Sleep Apnea: Meta-Analytic Review. J Med Internet Res 22(2):e16972 32130137 10.2196/16972 PMC7055847

[23] Chen C et al (2020) Telemonitor care helps CPAP compliance in patients with obstructive sleep apnea: a systemic review and meta-analysis of randomized controlled trials. Ther Adv Chronic Dis 11:2040622320901625 32215196 10.1177/2040622320901625 PMC7065282

[24] Anttalainen U et al (2016) Telemonitoring of CPAP therapy may save nursing time. Sleep Breath 20(4):1209–1215 27043327 10.1007/s11325-016-1337-9

[25] Cistulli PA et al (2019) Short-term CPAP adherence in obstructive sleep apnea: a big data analysis using real world data. Sleep Med 59:114–116 30799254 10.1016/j.sleep.2019.01.004 PMC6589354

[26] Chervin RD et al (1997) Compliance With Nasal CPAP Can Be Improved by Simple Interventions. Sleep 20(4):284–289 9231954 10.1093/sleep/20.4.284

[27] Fox N et al (2012) The impact of a telemedicine monitoring system on positive airway pressure adherence in patients with obstructive sleep apnea: a randomized controlled trial. Sleep 35(4):477–481 22467985 10.5665/sleep.1728 PMC3296789

[28] Turino C et al (2017) Management of continuous positive airway pressure treatment compliance using telemonitoring in obstructive sleep apnoea. Eur Respir J 49(2) 1601128: 1-8 10.1183/13993003.01128-2016 28179438

[29] Richard W et al (2007) Acceptance and long-term compliance of nCPAP in obstructive sleep apnea. Eur Arch Otorhinolaryngol 264(9):1081–6 17443336 10.1007/s00405-007-0311-3

[30] Susheel PP et al (2019) Treatment of Adult Obstructive Sleep Apnea With Positive Airway Pressure: An American Academy of Sleep Medicine Systematic Review, Meta-Analysis, and GRADE Assessment. J Clin Sleep Med 15(02):301–334 30736888 10.5664/jcsm.7638 PMC6374080

